# Awareness of the Causes Leading to Surgical Ablation of Ovarian Function in Premenopausal Breast Cancer—A Single-Center Analysis

**DOI:** 10.3390/medicina57040385

**Published:** 2021-04-16

**Authors:** Joana Correia Oliveira, Filipa Costa Sousa, Inês Gante, Margarida Figueiredo Dias

**Affiliations:** 1 Gynecology Department, Centro Hospitalar e Universitário de Coimbra, Praceta Prof. Mota Pinto, 3004-561 Coimbra, Portugal; inesrcgante@gmail.com (I.G.); marg.fig.dias@gmail.com (M.F.D.); 2 Clinical Academic Center of University of Coimbra, University Clinic of Gynecology, Faculty of Medicine, Azinhaga de Santa Comba, Celas, 3000-548 Coimbra, Portugal

**Keywords:** breast cancer, receptor, estrogen, salpingo-oophorectomy, antineoplastic agents, hormonal, neoplasms, hormone-dependent

## Abstract

*Background and Objectives*: Ovarian surgical ablation (OSA) in estrogen receptor-positive (ER+) breast cancer is usually performed to halt ovarian function in premenopausal patients. Since alternative pharmacological therapy exists and few studies have investigated why surgery is still performed, we aimed to analyze the reasons for the use of OSA despite the remaining controversy. *Materials and Methods*: Premenopausal ER+ breast cancer patients treated at a tertiary center (2005–2011) were selected, and patients with germline mutations were excluded. *Results*: Seventy-nine patients met the inclusion criteria. Globally, the main reasons for OSA included: continued menstruation despite hormone therapy with or without ovarian medical ablation (OMA) (34.2%), patient informed choice (31.6%), disease progression (16.5%), gynecological disease requiring surgery (13.9%), and tamoxifen intolerance/contraindication (3.8%). In women aged ≥45 years, patient choice was significantly more frequently the reason for OSA (47.4% versus 17.1% (*p* = 0.004)). For those aged <45 years, salvation attempts were significantly more frequent as compared to older women (26.8% versus 5.3% (*p* = 0.01)). In 77.8% of women undergoing OSA with menstrual cycle maintenance, surgery was performed 1–5 years after diagnosis, while surgery was performed earlier (0–3 months after diagnosis) in patients undergoing OSA as an informed choice (56.0%), as a salvation attempt (53.8%), or due to gynecological disease (63.6%). The leading reason for OSA in women previously undergoing OMA was continued menstruation (60.0%). *Conclusions*: This study suggests a possible failure of pharmacological ovarian suppression and reinforces the need for shared decision-making with patients when discussing treatment strategies, although validation by further studies is warranted due to our limited sample size.

## 1. Introduction

In premenopausal women with estrogen-receptor-positive (ER+) breast cancer, the higher levels of circulating 17β-estradiol (E2) represent a major risk factor for the recurrence or progression of tumors. This risk also exists in postmenopausal women, whose ovaries still produce some E2, although the main E2 source is from peripheral aromatase conversion of androgens into estrogens in adipose tissue [1]. In premenopausal breast cancer patients, bilateral oophorectomy (ovarian surgical ablation, OSA) markedly reduces the levels of circulating E2 to a point that some experts believe cannot be achieved by ovarian medical ablation (OMA) with gonadotrophin-releasing hormone analogs (GnRHa) [2]. GnRHa prevent follicular development and so reduce estrogen levels but, once suspended, there can be a rapid recovery of ovarian function [1,3]. In premenopausal low-risk ER+ breast cancer, the preferred therapeutic approach includes selective estrogen receptor modulators like tamoxifen, which have a greater affinity for breast ER than E2, and thus are able to displace them and bind themselves to the ER [1,4]. OSA or OMA are also more routinely performed in cases of premenopausal high-risk ER+ breast cancer [5]. Although tamoxifen can be used as monotherapy in low-risk patients, its combination with OSA or OMA is also considered an option [4,6]. In premenopausal metastatic or recurrent cases, therapy with OSA or OMA plus aromatase inhibitors (AIs) is standard [5]. An advantage of OSA is the concurrent reduction in ovarian androgen production and ovarian cancer risk [7]. Furthermore, if adjuvant AIs are used in combination with ovarian ablation to prevent extra-ovarian estrogen production, it is possible to achieve a blockade of peripheral and ovarian estrogen synthesis, reducing the circulating estrogen to the lowest level [8,9]. AIs can induce gonadotropin secretion due to a negative feedback effect on the hypothalamus [10]. For this reason, they should not be used if there is still ovarian function and should only be used in a menopausal state [2,10,11]. Some experts claim that OSA and OMA are similar in terms of efficiency, especially concerning early ER+ breast cancer [2,7,12]. The choice of one over the other varies according to patient compliance, costs, side effects, and possible complications [3]. As laparoscopic procedures have largely improved in recent years, the possibility of a minimally invasive surgical procedure has reinforced bilateral oophorectomy as a method for the permanent cessation of ovarian functions due to its relatively low rate of complications [7]. As there is no consensus concerning this approach, the patient’s preferences should also be considered in the decision-making process. Nevertheless, the selection of patients for either OSA or OMA in premenopausal ER+ breast cancer should be carefully considered. It should be recalled that these premenopausal women may still expect longevity and there is a risk of causing unnecessary long-term risks, mainly with respect to cardiovascular diseases. Moreover, we may also expose these women to decreased quality of life due to early menopausal symptoms and sexual dysfunction [13,14]. Since there is an alternative medical therapy (OMA) to reduce circulating levels of estrogen in premenopausal patients and few studies have investigated why surgery is still performed, in this article we aim to analyze why OSA is still performed despite the controversy that remains among experts in this field.

## 2. Materials and Methods

### 2.1. Study Design

This was a retrospective, descriptive, and inferential study conducted at the Gynecology Department of Coimbra Hospital and University Center, Portugal. Inclusion criteria: female patients with breast cancer diagnosed between 2005 and 2011, positive estrogen-receptor status confirmed by immunohistochemistry of the biopsy or surgical specimen of the lumpectomy or mastectomy, premenopausal status, and consent to undergo OSA. Exclusion criteria: presence of germline mutations such as breast cancer 1 and 2 (BRCA) and other cancer-related genes, and/or contraindications to or refusal of surgery. The patients’ clinical records were consulted after they had provided their informed written consent, which included a guarantee of anonymity.

### 2.2. Statistical Analysis

We created and performed statistical analysis of the database using The SPSS Statistics program version 26.0 (IBM Corp., Armonk, NY, USA). Descriptive statistics were analyzed as means and standard deviations for the variables with normal distribution, and as medians and interquartile ranges for the variables without normal distribution. The variables were described using absolute numbers (*n*). Frequencies and proportions were applied for categorical variables. Categorical data were analyzed by the chi-squared test or Fisher’s exact test. Statistical significance was set at *p* < 0.05.

## 3. Results

We selected 79 patients who fitted our selection criteria, which corresponded to 5.8% of the women with breast cancer treated at our center during the study period (*n* = 1356). General data relating to epidemiological, clinical, and pathological patterns are summarized in Table 1.

We have included the reasons that led to OSA, which were as follows: maintenance of ovarian function with menstrual cycles, patient choice after adequate information from the doctor and a finalized reproductive plan, disease progression/salvation attempt, organic gynecological disease, and contraindication or severe intolerance to hormone therapy with tamoxifen. The leading cause for OSA was maintenance of menstrual cycles (34.2% (*n* = 27)). Concerning the reasons for OSA according to age, in older women (≥45 years) patient choice was recorded as a significantly more frequent reason for OSA than in younger women (<45 years) 47.4% versus 17.1% (*p* = 0.004)). On the other hand, in younger women (<45 years), salvation was recorded as a significantly more frequent reason (26.8% versus 5.3% (*p* = 0.01)) than in older women. The reasons for OSA in both groups are described in Table 2.

In Table 3, epidemiological and clinical data according to groups of reasons for OSA are described.

In women submitted to OSA for retaining menstrual cycles, the majority (77.8%) had undergone this surgical procedure 1 to 5 years after diagnosis. On the other hand, surgery was performed earlier (0–3 months after diagnosis) in most women who underwent OSA for reasons of patient choice (56.0%), disease progression/salvation attempt (53.8%), and organic gynecological disease (63.6%). Analyzed data on the group of patients who had previously undergone OMA (*n* = 25) are presented in Table 4.

In the present study, the timeline between breast cancer diagnosis and OSA was also evaluated. In half of the patients who underwent immediate OSA (0 to 3 months after diagnosis) this method of ovarian ablation was their own choice. In all patients who underwent OSA after 5 years of breast cancer diagnosis, the surgery was performed due to the maintenance of menstrual cycles.

## 4. Discussion

Although breast cancer mostly occurs in postmenopausal women, 18–21% are diagnosed before menopause and 60% are hormone receptor-positive [15]. In ER+ tumors, tamoxifen, chemotherapy, ovarian surgical, or medical ablation and aromatase inhibitors are part of a possible adjuvant treatment strategy. In premenopausal women with hormone-dependent breast cancer, adjuvant OMA or OSA in association with other systemic therapies has been shown to significantly improve results with regard to recurrence and death after recurrence [16,17]. OSA has the advantages of reducing E2 production more extensively and irreversibly, reducing ovarian androgen production and lowering ovarian cancer risk. Vogl et al. advised that support for ovarian ablation is derived from studies that have many limitations, and that ovarian ablation should therefore not be performed except in situations with the highest risk [18]. The Suppression of Ovarian Function Trial (SOFT) and Tamoxifen and Exemestane Trial (TEXT), upon which the American Society of Clinical Oncology (ASCO) guidelines are based, selected post-chemotherapy patients only if they had functional ovaries and tested positive for hormone receptors, overcoming the limitations of previous studies [18]. In our study, all tumors were ER+, and the premenopausal status was confirmed in all patients. The ASCO guidelines recommend that higher-risk patients receive OSA or OMA combined with adjuvant endocrine therapy (either tamoxifen or AI), although there are no definitive criteria for defining the higher-risk cases [19]. According to the ASCO recommendations, target cases are stage II and III breast cancer patients who are usually proposed for chemotherapy, as well as stage I or II breast cancer patients with high recurrence risk and in whom chemotherapy is considered [19]. In our study, many patients were at high-risk: 91.1% (*n* = 72) patients had invasive ductal carcinomas (IDC) or IDC + ductal carcinoma in situ (DCIS); 11.4% (*n* = 9) were grade 3; 20.3% (*n* = 16) were human epidermal growth factor receptor type 2(HER2)-positive; 41.2% (*n* = 33) had axillary node invasion; and 13.9% (*n* = 11) were stage III or IV at diagnosis. These factors all supported the decision to carry out OSA. As shown in Table 1, most women who underwent OSA were still menstruating and maintained normal ovarian function despite hormone therapy with or without OMA (34.2%, *n* = 27). This observation supports the concern that E2 produced by the still-functional ovaries may interfere with the course of the disease, with more unfavorable outcomes. Considering the data in Table 2, the leading cause for OSA in older women (≥45 years) was patient choice (47.4%). Indeed, it would have been expected that younger women are more likely to maintain ovarian function and menstrual cycles after hormone therapy, but notably there was no statistically significant difference between the two groups. Moreover, there was a statistically significant difference regarding the age of patients in whom OSA was performed by choice and in those in which it was performed as a salvation attempt. It might be expected that older women would rather opt for a definitive procedure, as their ovarian function is already reduced and their reproductive plan is probably completed, and also that younger women tend to have more advanced/aggressive tumors, and our findings confirm these expectations. Globally, the second most frequent reason for OSA was patient choice (Group 2), and it was performed during the first year after diagnosis in 88.0% of cases, which probably reflects the inconvenience for patients of making regular visits to the hospital for therapeutic administration of goserelin, associated with a sense that OSA has greater reliability because it is irreversible after the advantages and disadvantages of each are properly explained by their physicians. Group 3 (salvation attempt) shows that in 16.5% of the patients, OSA was performed as an attempt to improve overall survival, as 84.6% of these patients had distant metastases at the time of diagnosis (mostly bone metastases). Five years after OSA, 61.5% (*n* = 8) of these metastatic patients were still alive, reflecting a possible benefit of OSA with regard to survival rates. In Group 4 (organic gynecological diseases) OSA was performed at the same time as the hysterectomy due to uterine pathology in 81.8% of patients (*n* = 9). As patients were to undergo a major surgical intervention, it was decided after patient consent to take the opportunity to perform the OSA procedure due to its advantages, with the intention of reducing the morbidity related to potentially undergoing two surgical procedures. In Group 5 (tamoxifen contraindications or severe intolerance), two patients had high cardiovascular risk which prevented the use of tamoxifen, and another patient suffered from one of the most common and serious side effects of tamoxifen, severe polyarthralgia [20], causing an unavoidable intolerance to this drug. Due to the especially small sample size of Groups 3, 4, and 5, and considering the inherent statistical limitations, these results should be carefully interpreted. More studies with larger samples are warranted to further elucidate the reasons for OSA.

In women who previously underwent OMA, the leading cause for OSA was, as expected, retaining menstrual cycles (60.0%, *n* = 15), which reflects a possible incomplete effect of ovarian ablation of goserelin, the GnRHa used in our patients. All 15 patients had been under hormone therapy with tamoxifen. In fact, the criteria for selecting either OSA or OMA are not well defined in the literature [3], and there is a lack of scientific evidence which makes the decision-making process difficult. Some experts believe that after OMA, the ovaries may still have a residual function [7,11,21]. Hagemann et al. suggested that the surgical approach is more cost-effective, mainly if treatment with goserelin is expected to last longer than 2 years [22]. Kwon et al. and Ferrandina et al. came to similar conclusions [7,11], finding that a laparoscopic bilateral salpingo-oophorectomy approach had a lower mean total cost and provided similar gains in quality-adjusted life years (QALYs) compared to a medical approach [7,11]. Moreover, it must be emphasized that OMA with GnRHa causes an initial flare in ovarian hormone production, which might have a detrimental effect on hormone-dependent breast cancer overall survival and disease-free survival. OSA also has the advantage of sparing patients from regular injections for 2 years [12]. The long-term consequences of iatrogenic menopause are rarely addressed in breast cancer literature. Various studies on this topic in non-breast cancer women refer to an increased mortality risk, but some of the studies only reported it after a 15-year follow-up period [23,24,25,26]. Reinforcing this concern, some of the consequences of premature menopause are usually only observed after 20 to 30 years [11]. OSA or OMA might be responsible for decreasing patients’ quality of life due to vasomotor symptoms, sexual dysfunction, psychological adverse effects, and an augmented risk of osteoporosis and fractures [3]. In addition, from a long-term perspective, coronary heart disease, stroke, and osteoporosis are all indicated as the main consequences of prematurely induced menopause [11]. According to the SOFT/TEXT trial conclusions, patients who had undergone OMA referred to posterior vasomotor symptoms, vaginal dryness, and decreased libido [20]. Pan Zhang et al., in their meta-analysis, assessed depression for the first time and did not find a significant difference; however, their results on vaginal dryness and hot flushes were consistent with the previous studies [15]. Nourmoussavi et al. suggested that a trial with goserelin in high-risk women undergoing OSA should be undertaken in order to evaluate patient adaptation to imposed menopause and its effect on quality of life. For those who want to go ahead with the treatment, the clinician could proceed with a definitive surgical strategy, always following a complete informed consent process with the patient about the long-term mortality risks [3]. One of our study’s limitations was the short follow-up time. Most breast cancer-related deaths and systemic relapses take 10–15 years to occur [18]. Trials like SOFT/TEXT used short-term follow-up periods as well, so definitive conclusions cannot be drawn [18]. In reality, there is a gap in the literature concerning long-term follow-up studies on multiple hormone adjuvant breast cancer therapies, so the literature does not account for long-term consequences and increased mortality risk related to iatrogenic menopause in this context. Other limitations of our study include its small sample size, the derivation of data from a single institution, and the differences between group sizes.

## 5. Conclusions

In our study, the leading cause of OSA was continued ovarian function with menstrual cycles despite previous hormone therapy with tamoxifen or OMA. The second most frequent reason for OSA was the informed choice of the patient, reflecting the need for a shared discussion taking the patients’ opinion into account and considering the advantages and disadvantages of the procedure at the time of the therapeutic strategy decision-making. Our study also suggests a possible failure of pharmacological ovarian suppression and reinforces that this should be considered when deciding which type of ovarian ablation to perform.

Though the long-term consequences of premature and irreversible iatrogenic menopause in ER+ breast cancer women probably encompass an increased mortality risk not causally related to the cancer, the advantages concerning improved disease-free survival and overall survival may outweigh this risk.

Definitive conclusions cannot be drawn from our findings due to the small number of cases, and further research on how to best address this field is needed, namely with prospective observational analysis with a larger study population and randomized clinical trials in order to better understand the future implications of this procedure.

## Figures and Tables

**Table 1:** Epidemiological, clinical, and pathological patterns (*n* = 79).

**Age (Median, IQR) Years**	43.5 IQR 7 (8–52)
<45 years	51.9% (*n* = 41)
≥45 years	48.1% (*n* = 38)
**BMI (Median, IQR) kg/m^2^**	24.5 IQR 6 (18–42)
**Relevant Clinical Background**	
Obesity (BMI > 30.0 kg/m^2^)	14.3% (*n* = 11)
Past thromboembolic events	1.3% (*n* = 1)
**Gynecological Data**	
Age of menarche (mean ± SD years)	12.2 ± 1.5 (9–16)
Combined hormonal contraception (any length)	48.1% (*n* = 38)
Combined hormonal contraception length of use >10 years	26.6% (*n* = 21)
**Obstetric Data**	
≥1 Term pregnancy	72.2% (*n* = 57)
Breastfeeding	59.5% (*n* = 47)
Duration in months (median, IQR)	6 IQR 8 (1–24)
**Reasons for OSA**	
Remaining menstrual cycles (Group 1)	34.2% (*n* = 27)
Patient choice (Group 2)	31.6% (*n* = 25)
Disease progression (Group 3)	16.5% (*n* = 13)
Gynecological organic disease (Group 4)	13.9% (*n* = 11)
Tamoxifen intolerance or contraindication (Group 5)	3.8% (*n* = 3)
**Tumor Characteristics**	
**Differentiation Grade**	
Well differentiated (Grade 1)	25.3% (*n* = 20)
Moderately differentiated (Grade 2)	63.3% (*n* = 50)
Poorly differentiated (Grade 3)	11.4% (*n* = 9)
**Histopathological Analysis**	
DCIS	8.9% (*n* = 7)
IDC	73.4% (*n* = 58)
IDC + DCIS	17.7% (*n* = 14)
**Immunohistochemistry**	
Positive estrogen receptors	100.0% (*n* = 79)
Positive progesterone receptors	88.6% (*n* = 70)
Positive HER2	20.3% (*n* = 16)
**Axillary invasion**	41.2% (*n* = 33)

Legend: BMI = body mass index; DCIS = ductal carcinoma in situ; HER2 = human epidermal growth factor receptor type 2; IDC = invasive ductal carcinoma; IQR = interquartile range; Kg = kilogram; M^2^ = square meter; OSA = ovarian surgical ablation; SD = standard deviation.

**Table 2:** Reasons for ovarian surgical ablation according to age <45 years (*n* = 41) vs. age ≥45 years (*n* = 38).

Reasons for Ovarian Surgical Ablation	<45 Years	≥45 Years	*p*
Maintenance of menstruation	41.5% (*n* = 17)	26.3% (*n* = 10)	0.156
Patient’s informed choice	17.1% (*n* = 7)	47.4% (*n* = 18)	0.004
Disease progression/salvation attempt	26.8% (*n* = 11)	5.3% (*n* = 2)	0.01
Organic gynecological disease	9.8% (*n* = 4)	18.4% (*n* = 7)	0.266
Contraindications/severe intolerance to hormone therapy with tamoxifen	4.9% (*n* = 2)	2.6% (*n* = 1)	1

**Table 3:** Epidemiological and clinical data according to groups of reasons for ovarian surgical ablation.

**Maintenance of Menstruation—Group 1 (*n* = 27)**	
**Age (Median, IQR)**	43.0 (IQR 6) years
<40 years	22.2% (*n* = 6)
40–45 years	48.1% (*n* = 13)
>45 years	29.6% (*n* = 8)
**Neoadjuvant chemotherapy**	11.1% (*n* = 3)
**Previous hormone therapy**	100.0% (*n* = 27)
**Previous OMA**	55.6% (*n* = 15)
**Time Between Diagnosis and OSA**	
0 to 3 months	0.0% (*n* = 0)
>3 months to 11 months	11.1% (*n* = 3)
1 year to 5 years	77.8% (*n* = 21)
>5 years	11.1% (*n* = 3)
**Hormone Therapy after OSA**	
Tamoxifen	48.1% (*n* = 13)
Aromatase inhibitors	37.0% (*n* = 10)
Switch to aromatase inhibitors after tamoxifen	3.7% (*n* = 1)
**Adjuvant radiotherapy**	66.7% (*n* = 18)
**Adjuvant chemotherapy**	40.7% (*n* = 11)
**Locoregional recurrence (until 2020)**	7.4% (*n* = 2)
**Distant metastasis (until 2020)**	3.7% (*n* = 1)
**5-year disease-free survival**	96.3% (*n* = 26)
**5-year overall survival**	100.0% (*n* = 27)
**Death from any cause (until 2020)**	11.1% (*n* = 3)
**Patient’s Informed Choice—Group 2 (*n* = 25)**	
**Age (Median, IQR)**	46.0 (IQR 5) years
<40 years	12.0% (*n* = 3)
40–45 years	28.0% (*n* = 7)
>45 years	60.0% (*n* = 15)
**Previous hormone therapy**	4.0% (*n* = 1)
**Previous OMA**	4.0% (*n* = 1)
**Time Between Diagnosis and OSA**	
0 to 3 months	56.0% (*n* = 14)
>3 months to 11 months	32.0% (*n* = 8)
1 year to 5 years	12.0% (*n* = 3)
>5 years	0.0% (*n* = 0)
**Locoregional recurrence**	4.0% (*n* = 1)
**5-year disease-free survival**	72.0% (*n* = 18)
**5-year overall survival**	88.0% (*n* = 22)
**Death from any cause (until 2020)**	12.0% (*n* = 3)
**Disease Progression/Salvation Attempt—Group 3 (*n* = 13)**	
**Age (Median, IQR)**	40.9 (IQR 6) years
<40 years	23.1% (*n* = 3)
40–45 years	69,2% (*n* = 9)
>45 years	7.7% (*n* = 1)
**Positive HER2** Adjuvant trastuzumab	15.4% (*n* = 2) 100.0% (*n* = 2)
**Tumor Grade**	
Grade 1	15.4% (*n* = 2)
Grade 2	84.6% (*n* = 11)
Grade 3	0.0% (*n* = 0)
**TNM Classification at Diagnosis (T)**	
2	53.8% (*n* = 7)
3	23.1% (*n* = 3)
4	23.1% (*n* = 3)
**TNM Classification at Diagnosis (N)**	
0	7.7% (*n* = 1)
1	53.8% (*n* = 7)
2	38.5% (*n* = 5)
**TNM Classification at Diagnosis (M)**	
0	15.4% (*n* = 2)
1	84.6% (*n* = 11)
**Local of Distant Metastasis**	
Bone	54.5% (*n* = 6)
Visceral	18.2% (*n* = 2)
Visceral and bone	27.3% (*n* = 3)
**Stage at Diagnosis**	
I or II	15.4% (*n* = 2)
III or IV	84.6% (*n* = 11)
**Neoadjuvant chemotherapy**	76.9% (*n* = 10)
**Axillary Lymph Node Dissection**	76.9% (*n* = 10)
During the first surgery	90.0% (*n* = 9)
After the first surgery (disease progression)	10.0% (*n* = 1)
**Pathological Characteristics**	
IDC	76.9% (*n* = 10)
DCIS	15.4% (*n* = 2)
IDC + DCIS	7.7% (*n* = 1)
**Previous hormone therapy**	30.8% (*n* = 4)
**Previous OMA**	30.8% (*n* = 4)
**Time Between Diagnosis and OSA**	
0 to 3 months	53.8% (*n* = 7)
>3 months to 11 months	23.1% (*n* = 3)
1 year to 5 years	23.1% (*n* = 3)
>5 years	0.0% (*n* = 0)
**Hormonal Therapy after OSA**	
Tamoxifen	15.4% (*n* = 2)
Aromatase inhibitors	76.9% (*n* = 10)
Switch to aromatase inhibitors after tamoxifen	7.7% (*n* = 1)
**Adjuvant radiotherapy**	28.0% (*n* = 7)
**Adjuvant chemotherapy**	61.5% (*n* = 8)
**5-year disease-free survival**	23.1% (*n* = 3)
**5-year overall survival**	61.5% (*n* = 8)
**Death from any cause (until 2020)**	69.2% (*n* = 9)
**Organic Gynecological Disease—Group 4 (*n* = 11)**	
**Age (Median IQR)**	47.4 ± 4.8 (IQR 13) years
<40 years	0.0% (*n* = 0)
40–45 years	45.5% (*n* = 5)
>45 years	54.5% (*n* = 6)
**Organic Gynecological Disease**	
Myomatous uterus	(*n* = 6)
Hysterocele	(*n* = 2)
Premalignant endometrial disease	(*n* = 1)
Bilateral ovarian complex cysts	(*n* = 2)
**Previous hormone therapy**	18.2% (*n* = 2)
**Previous OMA**	18.2% (*n* = 2)
**Time Between Diagnosis and OSA**	
0 to 3 months	63.6% (*n* = 7)
>3 months to 11 months	9.1% (*n* = 1)
1 year to 5 years	27.3% (*n* = 3)
>5 years	0.0% (*n* = 0)
**Locoregional recurrence**	0.0% (*n* = 0)
**5-year disease-free survival**	100.0% (*n* = 11)
**5-year overall survival**	100.0% (*n* = 11)
**Death from any cause (until 2020)**	0.0% (*n* = 0)
**Contraindications/Severe Intolerance to Tamoxifen—Group 5 (n = 3)**	
**Age**	
<40 years	33.3% (*n* = 1)
40–45 years	33.3% (*n* = 1)
>45 years	33.3% (*n* = 1)
**Contraindications or Intolerance to Tamoxifen**	
Significant cardiovascular risk	(*n* = 2)
Severe neurologic symptoms and polyarthralgia	(*n* = 1)
**Previous hormone therapy**	100.0% (*n* = 3)
**Previous OMA**	100.0% (*n* = 3)
**Locoregional recurrence**	33.3% (*n* = 1)
**5-year disease-free survival**	33.3% (*n* = 1)
**5-year overall survival**	100.0% (*n* = 3)
**Death from any cause (until 2020)**	0.0% (*n* = 0)

Legend: DCIS = ductal carcinoma in situ; HER2 = human epidermal growth factor receptor type 2; IDC = invasive ductal carcinoma; IQR = interquartile range; OMA = ovarian medical ablation; OSA = ovarian surgical ablation.

**Table 4:** Patients who had previously undergone ovarian medical ablation (*n* = 25).

**Age (** **Mean ± SD)**	40.8 ± 5.3 (28–52) years
<45 years	80.0% (*n* = 20)
≥45 years	20.0% (*n* = 5)
**Reasons for OSA**	
Maintenance of menstruation	60.0% (*n* = 15)
Patient informed choice	4.0% (*n* = 1)
Disease progression/salvation attempt	16.0% (*n* = 4)
Organic gynecological disease	8.0% (*n* = 2)
Contraindications/severe intolerance to tamoxifen	12.0% (*n* = 3)
**Hormone therapy with tamoxifen**	88.0% (*n* = 22)

Legend: OSA = ovarian surgical ablation; SD = standard deviation.

## Data Availability

The data presented in this study are available on request from the corresponding author.
